# Formulation, stability, and delivery of bacteriophage cocktails in veterinary medicine: a review of physicochemical constraints and therapeutic outcomes in production animals

**DOI:** 10.3389/fvets.2026.1988130

**Published:** 2026-10-15

**Authors:** Muhammad Muneeb, Mohamed Tharwat, Aftab Shaukat, Nourhan Nassar, Yonggang Qu, Asfand Yar Khan, Fahad A. Alshanbari

**Affiliations:** 1 College of Animal Science and Technology, Shihezi University, Shihezi, China; 2 Department of Clinical Sciences, College of Veterinary Medicine, Qassim University, Buraidah, Saudi Arabia; 3 College of Veterinary Medicine, Huazhong Agricultural University, Wuhan, China; 4 College of Veterinary Medicine, South China Agricultural University, Guangzhou, Guangdong, China; 5 Department of Clinical Sciences, Faculty of Veterinary Sciences, The University of Veterinary and Animal Sciences, Swat, Pakistan; 6 Department of Medical Biosciences, College of Veterinary Medicine, Qassim University, Buraidah, Saudi Arabia

**Keywords:** antimicrobial resistance, bacteriophage cocktails, formulation strategies, livestock, physicochemical barriers, treatment outcome, veterinary phage therapy

## Abstract

Intensive food animal production is at risk due to the ever-increasing antimicrobial resistance (AMR) of veterinary pathogens, and it is time to look at alternatives to antibiotics. Bacteriophage (phage) cocktails, which involve the use of several bacteriophages with different host ranges and resistance profiles, are a new and exciting biologic approach. Nevertheless, there are significant physicochemical barriers in the animal host and during feed processing that hinder the translation of promising *in vitro* bacteriolysis to consistent *in vivo* therapeutic efficacy. The review critically addresses the interrelated issues of gastric acidity, enzymatic digestion, exposure to bile salts, thermal stress during feed pelleting and osmotic fluctuations, which all contribute to the destruction of orally administered phage titers. We systematically examine formulation technologies such as lyophilization, spray-drying, and alginate, chitosan, lipid nanoparticles and hydrogel encapsulation to protect phages from these destructive environments. When comparing the delivery routes of oral, parenteral, intramammary and aerosol, it is observed that each route places different priorities on biopharmaceutical requirements. Critical evaluation of therapeutic outcomes in poultry, swine, ruminants, and aquaculture has shown that the use of formulations optimized for the gastrointestinal physiology of the target species can lead to significantly reduced target bacterial loads and mortality rates, but failures often occur when *in vitro* stability data is not supported by an understanding of the pharmacokinetic parameters. For ruminants, there is a remarkable difference between the success of acid-resistant encapsulation in monogastrics and the inability of such methods to work in ruminants due to premature release and inactivation of phages by the forestomach microbiome. We emphasize the need for standardized assays of phage titer in different laboratories, well-defined regulatory processes, and physiologically-based pharmacokinetic/pharmacodynamic models that can predict the temporal and spatial dynamics of phage-bacteria interactions *in vivo*. This synthesis provides a roadmap for the rational design of phage cocktail products that can reliably reduce antibiotic dependence in veterinary medicine.

## 1 Introduction

The global intensification of poultry, swine, bovine, and aquaculture production has been accompanied by a steep increase in the use of antimicrobials for metaphylaxis, growth promotion, and treatment of infectious diseases. This selective pressure has accelerated the emergence and dissemination of multidrug-resistant (MDR) zoonotic and animal-specific pathogens, including extended-spectrum β-lactamase-producing *Escherichia coli*, methicillin-resistant *Staphylococcus aureus* (MRSA) in swine and dairy cattle, fluoroquinolone-resistant *Campylobacter* spp. in broilers, and *Vibrio* spp. harboring plasmid-mediated resistance in shrimp farming (1–3). Recognizing that antimicrobial resistance (AMR) threatens not only animal health but also human food safety and global public health, international bodies such as the WHO, OIE, and FAO have called for drastic reductions in veterinary antibiotic consumption and the development of effective alternatives (4, 5).

One of the most promising biological interventions are bacteriophages, viruses that specifically infect and lyse bacteria. Today, with the help of modern molecular biology and synthetic biology tools, the historical roots of the use of phages as therapy are revived as laid by d’Herelle in the early 20th century (6). Food-animal medicine presents a number of intrinsic advantages for the use of phages: they multiply in the area of infection, they are not harmful to mammalian cells, they can break down biofilms, and they are highly host-specific, which means that they have minimal impact on commensal flora (7). In most cases, however, monophage preparations will result in the rapid emergence of phage-resistant bacterial mutants, as observed in the models of *Salmonella*, *Campylobacter*, *E. coli* and *Aeromonas* (8, 9). Phage cocktails, which consist of a combination of several phages targeting different receptors or using different infection strategies, significantly lower the chances of the development of phage resistance and expand the range of host strains of circulating field strains (10). Thus, regulatory talks and commercial development projects are shifting to more well-defined cocktails instead of individual products per phage (11).

Although there is a lot of *in vitro* data showing that the cocktails are very effective at killing bacteria, the clinical effectiveness of the oral application of the cocktails in production animals is inconsistent (12). This is because phages are proteinaceous nanoparticles which are highly susceptible to the harsh physicochemical conditions that exist during storage, feed processing and gastrointestinal transit. The number of viable phages can drop by several orders of magnitude within minutes due to gastric acidity (pH < 3), pepsin-mediated proteolysis, pancreatic trypsin, a high temperature during pelleting (70–90 °C) and osmotic shocks in the drinking water delivery system (13, 14). Furthermore, the prandial state of the animal, digestive physiology and the dosage form have a significant impact on the proportion of the dose that reaches the target site in an active form (12). The main argument of this review is that to close the *in vitro*–*in vivo* gap in therapeutic efficacy in the treatment of bacterial infections requires a systematic approach that combines the biology of phages with the science of pharmaceutical formulation and species-specific pharmacokinetics.

The following sections present a comprehensive, critical analysis of: (i) physicochemical barriers to phages *in vivo* and *ex vivo*; (ii) formulation technologies designed to overcome these barriers; (iii) the effects of the delivery route on the formulation requirements; (iv) therapeutic outcomes in poultry, swine, ruminants, and aquaculture, focusing on the correlation of formulation characteristics and clinical endpoints; and (v) what remains to be done in scale-up, regulation, resistance management, and assay standardization. The entire pipeline for development of a bacteriophage cocktail is plotted in Figure 1 from the isolation of the phages to their application in the field, in eight sequential steps discussed throughout this review. Differences between high *in vitro* stability and *in vivo* success are emphasized throughout and the reasons for the failure of some encapsulation methods in the ruminant forestomach are explained.

**Figure 1:**
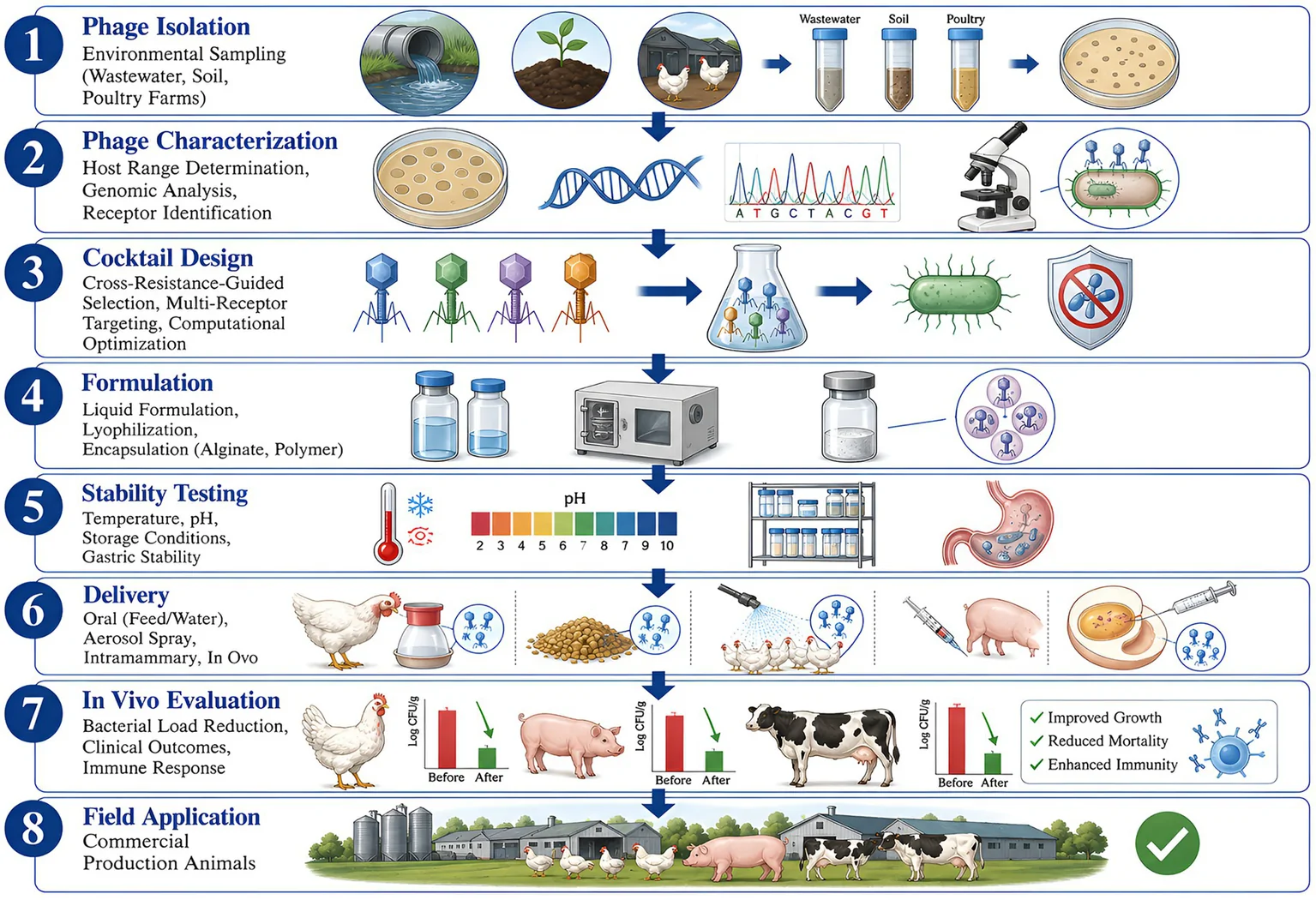
Schematic overview of the pipeline of developing a bacteriophage cocktail from isolation to application in the field. The eight sequential steps for the development of phage cocktails for veterinary use are shown in the flowchart: (1) Phage isolation from environmental samples (wastewater, soil, poultry farms); (2) Phage characterization including host range determination, genomic analysis, and receptor identification; (3) Rational cocktail design, employing cross-resistance-guided selection, multi-receptor targeting, and computational optimization; (4) Formulation, including liquid, lyophilized, or encapsulated (alginate/polymer); (5) Stability testing under various temperature, pH, storage, and gastric conditions; (6) Delivery, via oral (feed/water), aerosol spray, intramammary, or in ovo; (7) *In vivo* evaluation, assessing bacterial load reduction, clinical outcomes, and immune responses; and (8) Field application in commercial production animals. The sequential progression through each stage of development is shown by the blue arrows.

## 2 Physicochemical hurdles to phage cocktail viability *in vivo* and *ex vivo*

### 2.1 Gastric acidity and pH gradients

The first big challenge to phages that are given orally is the low pH in the stomach. Gastric pH is as low as 1.5–2.5 in fasted pigs and poultry (15) and 4.0–5.0 in fed animals, due to the buffering effect of feed (16, 17). Structural proteins of phages, including tail fibers and capsid subunits, are irreversibly denatured and aggregated at pH levels below 3.0, resulting in a rapid decrease in infectivity (18). The effect of exposure to simulated gastric fluid (SGF) (pH 2.5) for 30- to 60-min periods has been shown to decrease the number of *Salmonella* phages by 4–7 log_10_ PFU/mL (19). The stability of five coliphages (four of the family *Myoviridae* and one of the family *Siphoviridae*) was evaluated and it was found that the *Myoviridae* phages were stable in a pH range between 2.0 and 7.0, while the *Siphoviridae* phage (JK16) was highly sensitive to low pH conditions (20). There is known significant interspecies and interstrain variation in acid tolerance. For instance, *S. aureus* phages were found to be stable over the pH range 3.0 to 11.0 (21) and were completely inactivated at pH 3.0 after 24 h (22) with partial inactivation observed at intermediate pH levels. The receptor-binding protein structure and the presence of phase-variable modifications of the capsular polysaccharide (e.g., O-methyl phosphoramidate (MeOPN)) are critical phage receptors in *Campylobacter* phages and known to strongly influence survival and host range (23, 24). This variability highlights the importance of characterizing the acid sensitivity of each phage in a cocktail separately prior to the formulation design.

The pH gradient from the stomach to the small intestine is not a discrete step but a dynamic continuum. The pH in the duodenum quickly increases to 6.0–6.5 because of the secretion of bicarbonate, which can renature some phages if the denaturation was not followed by proteolytic cleavage (25). In an *in vitro* gastrointestinal model (TIM-1), phages were shown to be able to survive the acidic stomach environment and be delivered to the ileal compartment, with up to 27% of the initial phage dose recovered in the ileal compartment when suspended in a protective matrix like milk concentrate (26). A fraction of the dose can thus be saved using formulation strategies that will delay the release of the phages until the pH is above 5.5.

### 2.2 Enzymatic degradation (pepsin, trypsin, pancreatic enzymes)

Proteolytic enzymes in the stomach (pepsin) and small intestine (trypsin, chymotrypsin, elastase) are a major problem for phage proteins (27). Pepsin, whose optimum pH is 2, can break exposed peptide bonds and can rapidly break down the capsid and tail proteins of phages, even at low levels of acid-induced denaturation (28). *In vitro* experiments with simulated intestinal fluid (SIF) containing trypsin have shown that many *E. coli* and *Salmonella* phages lose 3–5 log_10_ of viability within 1–2 h (14, 29). The effect of pepsin on the viability of phages has been reported: Dini et al. (30) showed that phages encapsulated in alginate were protected from proteolytic degradation when challenged with simulated gastric conditions (pH 2.5, 3.2 mg/mL pepsin). Non-encapsulated phages, on the other hand, are completely inactivated in 10 min at pH 1.6 and with pepsin concentrations as low as 0.5 mg/mL (30). The enzyme may be encapsulated within a polymeric matrix, which may sterically hinder the approach of the enzyme to the phage surface, and the size of the pores in hydrogels is usually in the range of 5–200 nm, depending on the degree of crosslinking (31). The polymers, excipients, and crosslinking agents used have an impact on the protection of phages from gastric acid and digestive enzymes (32).

### 2.3 Bile salts and surfactant effects

In the fed animal, the bile salts secreted into the duodenum can reach concentrations of up to 20 mM, and are amphipathic molecules that can disrupt lipid membranes and denature proteins. With a few exceptions, including some species in the *Corticoviridae* (33, 34), phages are not covered with a lipid envelope, but bile salts can still have a profound effect on the viability of phages. For instance, free bacteriophage Felix O1 reduced by 1.29 and 1.67 log units after 3 h in 1 and 2% bile solutions, respectively (35). The scanning electron microscope revealed a significant degree of aggregation of the phages and tail sheath contraction, which is irreversible structural damage (36). Interestingly, phages with a higher surface charge or hydrophilicity (PEGylation), exhibit enhanced resistance to bile inactivation (37). Surface plasmon resonance (SPR) experiments showed that the binding affinity of PEGylated phages to bile acid micelles was 10-fold lower than that of native phages (37, 38). This observation has prompted interest in surface-modified phages or bile-salt-sequestering excipients for oral formulations. At the same time, dietary fibers like β-glucan are known to bind bile salts in the intestinal lumen (39, 40). This property is well known for cholesterol-lowering properties, but the ability of this property to protect the co-administered phages from inactivation by bile needs to be studied. Nevertheless, β-glucan has been proven to be a biocompatible matrix for the formulation of phages and their viability is maintained at >99% during storage (41).

### 2.4 Mucosal trapping and immune clearance

In addition to chemical degradation, phages are faced with physical obstacles like the mucus layer and the innate immune system of the host. Intestinal mucus, which consists of mucin glycoproteins, can trap phage particles by adhesive interactions, which will decrease the number of phages reaching the intestinal surface and, in turn, the intestinal lumen (42). This “mucosal entrapment” is especially important for non-motile phages that are passive diffusers. Notably, certain phages display Ig-like domains that promote adherence to mucin, a phenomenon proposed as a non-host-derived antimicrobial defense mechanism (43). However, for therapeutic phages, this binding can lead to a decrease in the effective concentration in the lumen. Recently, a porcine intestinal organoid model revealed that mucus-associated phages were removed from the intestinal lumen at 3–5 times the rate of non-mucoadhesive phages, which reduced the availability of subjacent intestinal pathogens (44). To counteract this, phage display engineering has been investigated to weaken mucin-binding whilst retaining the ability to bind to the receptor of the target bacteria.

Phages injected into the body are rapidly eliminated by the mononuclear phagocyte system, mainly in the liver and spleen. The liver has been found to contain 70–90% of the phages, and it is the Kupffer cells that quickly inactivate the phages; the spleen was found to contain the highest titres of phages and to contain active phages for the longest time (45, 46). This fast clearance requires high initial titres or repeated doses in systemic infections.

Additional challenges are repeated intravenous dosing. Tan et al. (47) showed that repeated IV phage administration in rats and monkeys led to a 2–3 log_10_ reduction in plasma phage titer 5 min after the dose, and to splenic enlargement and the induction of phage-specific neutralizing antibodies. Immune response is route dependent, the highest and most lasting antibody response was obtained after intraperitoneal administration, and the subcutaneous administration produced weaker and delayed antibody response (48).

PEGylation has been extensively explored as a strategy to prolong phage circulation in the body (49). Recent studies have demonstrated that the attachment of 5 kDa PEG chains to phage capsids can modify more than 60% of the surface without impairing replication competence. PEG-modified phages are more stable in blood serum, persist longer within the macrophages and remain in circulation longer in healthy and infected mice. Also, these PEGylated phages cause much lower levels of anti-phage antibodies compared to normal phages and help reduce harmful inflammation markers like IL-6, TNF-α, IFN-γ, and IL-1β to normal levels. However, other techniques, such as the use of poly(2-oxazoline) or zwitterionic coatings should be considered due to the possibility of developing antibody against PEG in some individuals (49).

### 2.5 Temperature fluctuations during feed pelleting and storage

For poultry and swine, steam conditioning and pelleting are standard methods for processing feed, typically between 70–90 °C. The majority of tailed phages (order *Caudovirales*) are extremely temperature sensitive, and high temperatures during processing are a significant formulation challenge (50). This significant heat sensitivity is a significant challenge for using phage cocktails in pelleted feeds unless protective formulations are used. Phages can become inactive over time in liquid feed or drinking water systems at farm ambient temperatures. The viability of lyophilized phage powders stored at 4 °C is generally months, and Manohar and Ramesh (51) showed the viability of phage cocktails at 4 °C and 37 °C to be months and 10 months, respectively, when lyophilized with sucrose-gelatin excipients. However, in resource-limited farming environments, cold-chain logistics remain a major challenge (52).

### 2.6 Osmotic and ionic strength variations in water and buffers

Phage stability is highly dependent on the ionic environment. The ionic environment plays a very critical role in phage stability. Some phages need divalent cations (Ca^2+^, Mg^2+^) to adsorb and inject their genome into the bacteria, and hypotonic shock can lead to the disruption of the capsid. Leibo and Mazur (53) showed that bacteriophages T4B and T4D undergo osmotic shock and are very sensitive to low monovalent salt concentrations, but there is strain-specific variation in sensitivity. Almost half of the osmotically shocked phages had their DNA intact, but they were no longer infective, suggesting that the phages suffered from structural damage (53).

The hardness and salinity of the drinking water provided to farms is variable and can cause osmotic lysis in the case of ultrapure water, and aggregation and loss of infectivity in the case of high salinity. Phages should be stabilized with standard laboratory buffers, such as SM buffer (100 mM NaCl, 10 mM MgSO_4_, 50 mM Tris–HCl, pH 7.5, 0.01% gelatin). It has been demonstrated that SM buffer at 4 °C can keep the infectivity of phages for up to 30 days and DNA for up to 100 days (54). However, such buffers are not economically viable for water medication systems of large scale.

Other excipients such as trehalose and skim milk powder also act to buffer against osmotic stress and are essential for the development of drinking water–stable formulations. Xu et al. (55) showed that skim milk powder along with trehalose reduced the extent of phage inactivation during lyophilization and subsequent storage at 28 °C. Kim et al. (56) prepared a stabilizing solution using sugars and salts, which greatly enhanced the survival of the phages in distilled water. Skim milk powder probably forms a protective protein corona around the phage particles, which is why it is able to preserve the titer even in water with low ionic strength.

### 2.7 Impact of prandial state (fed vs. fasted)

Gastric residence time, pH and enzyme secretion are significantly altered in the prandial state. In fasted animals, the pH of the stomach may reach 1.5–3.5, which is a very harsh environment for phages to survive in (57). On the other hand, the food matrix may protect the phages from the gastric acidity and act as physical protection during gastric transit. In dynamic gastrointestinal models, the protective effect of food matrices has been shown. Samtlebe et al. (26) used TIM-1 to show that a lactococcal phage P008 was able to survive in high numbers (>90%) after passing through the upper human gastrointestinal tract, and that high numbers of phages were delivered from the ileal compartment after 4 h of digestion. Interestingly, by delivering up to 27% in the ileal compartment, phages in milk concentrate appeared to be protected by the dairy matrix (26).

The significance of protective formulations for oral phage delivery has been confirmed by other studies. Phages were encapsulated in alginate with milk proteins, which protected phages from the acidic conditions (pH 2.5) for 2 h, and released in 30 min in intestinal conditions (58). Likewise, Verthé et al. (59) showed that at the gastric pH of 2.0, phage UZ1 was completely inactivated, whereas no significant changes in phage titer were seen after 2 h incubation at 37 °C if an antacid was used to neutralize the stomach acidity.

Smith et al. (60) had direct evidence that the interaction of prandial state, feed composition and phage survival occurred in calves. Phages were found in the small intestine at high levels (6.5–6.8 log PFU/g contents) and in the abomasum (pH 4.4) 0.3 h after phages were administered in milk to fasted calves. However, by 5 h after feeding (when the pH in the abomasum was 1.8), there was a significant reduction in phage titers; some phages were not detectable in the abomasum and lower titers in the small intestine. Phage survival during gastrointestinal transit was improved and gastric pH was maintained by the addition of CaCO_3_ to milk (60).

The interplay between formulation, meal timing, and gastric physiology must be empirically defined for each target species, since the gastric physiology of fed and fasted animals and across species will differ. From these results, it is recommended that oral phage cocktails be used in protective food matrices or with the use of encapsulation techniques that will protect the phages from the acidic environment of the stomach. The above physicochemical barriers, the basic mechanism of phage action and strategies to overcome resistance are summarized in Figure 2. The key physicochemical barriers to phage viability in the gastrointestinal tract, along with their corresponding mitigation strategies, are summarized in Table 1.

**Figure 2:**
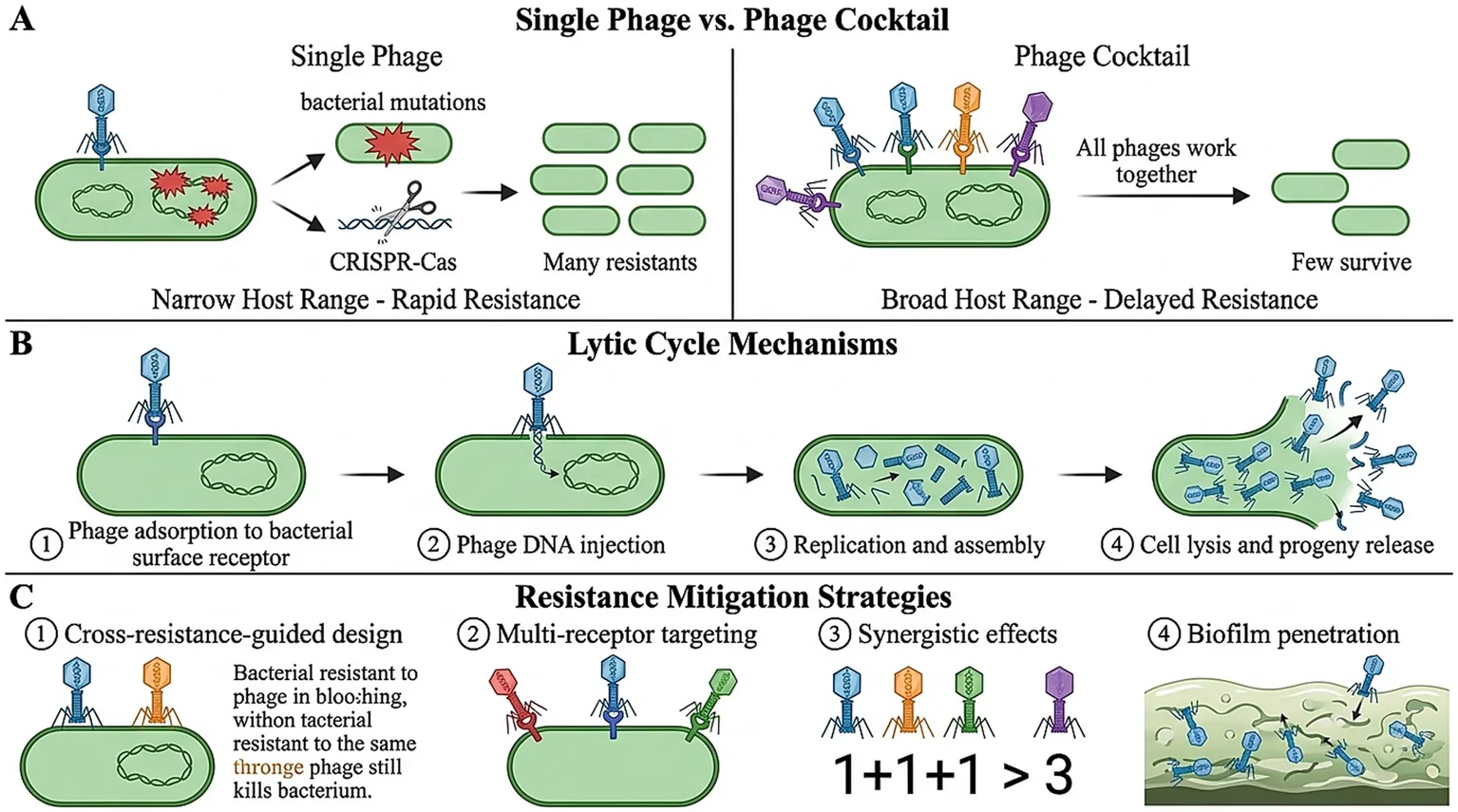
Mechanisms of bacteriophage cocktail action and resistance mitigation. **(A)** Comparison of single phage versus phage cocktail therapy. Single phages exhibit narrow host range and are susceptible to rapid resistance development through bacterial mutations and CRISPR-Cas systems, whereas phage cocktails provide broad host range through multiple receptors targeting and delayed resistance emergence. **(B)** The bacteriophage lytic cycle illustrating the four sequential steps of phage-mediated bacterial killing: (1) adsorption to bacterial surface receptors, (2) phage DNA injection, (3) intracellular replication and assembly of new phage particles, and (4) bacterial cell lysis and release of progeny phages. **(C)** Key resistance mitigation strategies employed in rational phage cocktail design: cross-resistance-guided design (resistance to one phage does not confer resistance to another), multi-receptor targeting (phages bind distinct bacterial surface receptors), synergistic effects (combined activity exceeds individual activities), and biofilm penetration and disruption.

**Table 1:** Physicochemical barriers to phage viability in the gastrointestinal tract and mitigation strategies.

Barrier	Relevant site(s)	Effect on phages	Formulation mitigation strategy	Key references
Gastric acidity (pH 1.5–2.5 fasted)	Stomach (monogastrics), abomasum (ruminants)	4–7 log_10_ loss in 30–60 min; capsid denaturation, tail fiber unfolding	Enteric coatings (Eudragit®), alginate–chitosan encapsulation, fed-state administration, milk-based buffers	(19, 20, 57)
Pepsin (pH 1.5–2.5)	Stomach	Synergistic with acid; cleaves capsid and tail proteins; non-encapsulated phages inactivated at 0.5 mg/mL pepsin within 10 min	Dense hydrogel matrices (pore size 5–200 nm), alginate encapsulation, Ca^2+^-stabilized capsules	(27, 30, 31)
Bile salts (≤20 mM)	Duodenum	1.29–1.67 log_10_ loss in 3 h (1–2% bile); capsid hydrophobic patch binding, aggregation, tail sheath contraction	PEGylation, hydrophilic surface coatings, alginate–chitosan encapsulation, β-glucan as bile-salt adsorbent	35–37
Trypsin/Chymotrypsin	Small intestine	3–5 log_10_ loss in 1–2 h in SIF; proteolytic degradation of capsid proteins	Encapsulation with enzyme-impermeable matrices, alginate–chitosan beads, PEGylation	(29, 31)
Temperature (pelleting: 70–90 °C)	Feed mill	>5 log_10_ loss in 2 min at 70 °C for most *Caudovirales*; capsid proteins denature at 55–60 °C	Spray-dried microcapsules with high-Tg excipients (trehalose, whey protein), isolation of thermostable phages, cold pelleting	(50, 71)
Osmotic/ionic stress (drinking water)	Farm water lines	Hypotonic shock causes capsid rupture; low-mineral water (<50 ppm TDS) causes titer loss; high salinity (>1% NaCl) promotes aggregation	Addition of SM buffer salts (MgSO_4_, NaCl), skim milk powder (2–5%), trehalose, sugar/salt stabilizing solutions	(53, 54, 56)
Mucosal trapping	Intestine, lung	Reduced luminal phage concentration; mucus-associated phages cleared 3–5 × faster than non-mucoadhesive phages	Engineering of tail fibers to reduce mucus binding; selection of non-mucoadhesive phage variants	(42–44)
Residual chlorine (0.5–2.0 ppm)	Drinking water	1–3 log_10_ inactivation within 30 min	Sodium thiosulfate, skim milk powder to quench chlorine; sugar/salt stabilizing solutions	(56)
Mononuclear phagocyte system (MPS) clearance	Liver, spleen	70–90% of phage particles concentrate in liver; plasma half-life of minutes in large animals; 2–3 log_10_ decline upon repeated IV dosing	PEGylation (>60% surface modification), serial-passage (long-circulating mutants), liposome encapsulation	(45–47, 49)
Antibody-mediated neutralization	Systemic circulation	Free phages neutralized within 3 h of antibody interaction; 38.5% immune neutralization in patients	Liposome encapsulation (100% protection), PEGylation (lower anti-phage IgG), intranasal delivery (avoids systemic antibodies)	(76, 84, 90, 145)

## 3 Formulation strategies for enhancing phage stability

### 3.1 Liquid and lyophilized bulk formulations (excipients and buffers)

Liquid formulations stabilized with buffer salts, sugars and proteins are the simplest phage formulations. Phage can survive in tris-buffered saline containing appropriate stabilizers but this suspension provides little protection against gastric acidity or heat stress. It has been reported that the presence of SM buffer (Tris/NaCl/MgSO_4_) can greatly enhance the survival of phages during the dehydration process (61). Phage T7 in SM buffer formulation also exhibited a low level of loss of titer of 0.19 log PFU during thin film freeze-drying, suggesting good preservation of phage function (61).

For long-term storage, lyophilization (freeze-drying) in the presence of cryoprotectants like trehalose, sucrose, skim milk and mannitol is now the standard method. Trehalose is particularly known to form an amorphous glass that protects the phage particles from denaturation by replacing water molecules through hydrogen-bonding (62, 63). Manohar and Ramesh (51) also found that sucrose, gelatin and their mixture were beneficial in retaining the viability of the phage cocktails after lyophilization and that the viability of the cocktails was retained for up to 10 months during storage at 4 °C.

But lyophilized cakes are hygroscopic and require to be packaged with desiccants and the reconstitution process may not be feasible in large-scale feed mills. Furthermore, lyophilization does not provide gastric acid protection and the lyophilized product must be further coated for oral delivery.

### 3.2 Encapsulation and micro/nano-particulate systems

#### 3.2.1 Alginate and chitosan-based systems

Calcium alginate hydrogels are among the most widely investigated encapsulants for phages because of their mild gelation conditions (ionic crosslinking by Ca^2+^), biocompatibility, and pH-responsive swelling. Alginate-based hydrogels help keep phages safe from tough environmental conditions like low pH and enzymes breaking them down, which helps maintain their ability to infect and work effectively as therapy (64). Phage release from the hydrogels occurs through a swelling–collapse–degradation process influenced by environmental factors such as pH, allowing sustained delivery over extended periods. The gelation mechanism involves ionic cross-linking with calcium ions, forming a three-dimensional network that can be fine-tuned by adjusting the alginate concentration and cross-linking density (64).

Several studies have documented the protective effect of alginate encapsulation against gastric conditions. A systematic comparison of alginate-based encapsulation strategies reported that alginate–chitosan beads with a core/shell structure allowed *E. coli* phage ZCEC5 to resist acidic environments (pH 2) for 60 min with only a 1 log_10_ reduction in embedded phage titer, while achieving complete phage release within 5 h under simulated intestinal fluid (SIF) conditions (65). Similarly, alginate–chitosan microspheres encapsulating *E. coli* phage O157: H7 showed resistance to pH 2 for 60 min with a 1.5 log_10_ reduction, with 65% of embedded phage released after 6 h in SIF (65). Importantly, polycationic coatings such as chitosan and polyethyleneimine (PEI) on alginate beads significantly increased acid resistance; alginate-only beads showed a 4 log_10_ reduction at low pH, while alginate–PEI beads showed only a 1 log_10_ reduction (65).

For *Salmonella* phages, alginate–chitosan beads containing phage Felix O1 demonstrated near-complete phage release (1.6 × 10^7^ PFU) within 5 h under SIF conditions, although exposure to SGF at pH 2.4 for 60 min resulted in a 2.6 log_10_ reduction in phage titer. At pH 2, no phage titer was observed after 30 min for this formulation (65). In comparison to alginate-only microspheres, Tang et al. (66) showed that alginate–whey protein microspheres greatly increased phage survival in simulated gastric fluid; free phage was totally inactivated in minutes in SGF, whereas encapsulated phage largely retained viability after two hours of incubation.

Combination encapsulations have been demonstrated to be effective for extended *in vivo* phage activity. Kim et al. (67) have created PLGA/alginate-composite microspheres that allow for phage detection in tissues for up to 28 days after administration, whereas non-encapsulated phages have been detected for only 3–5 days. The controlled release was blamed for reduced immune responses and thus enhanced prophylaxis.

Other polysaccharide mixtures have also been tested. Alginate–carrageenan and alginate–chitosan formulations have been proven to have efficiency in encapsulating phages and protecting them against acidic environment (68). In acidic environments, the recovery rates of phages in ALG-CG-CA were significantly higher than those in ALG-CG and unencapsulated phages, and the duration of phage activity and the protection uniformity were also significantly improved (69).

Although these are encouraging *in vitro* findings, *in vivo* efficacy will need to be considered. However, the use of chitosan has its drawbacks because conventional chitosan needs to be solubilized in acetic acid which affects the survivability of the phages (68). The fed state, gastric motility and interactions with feed components may affect the performance of encapsulated phages and *in vivo* testing is required under conditions that replicate the *in vivo* conditions of the target animal. This difference between *in vitro* and *in vivo* results highlights the need for dynamic dissolution models that mimic the fed-state gastric environment, fluid shear and intestinal peristalsis.

#### 3.2.2 Spray-dried microcapsules and powder formulations

Spray-drying is a promising and scalable process for making phage powders which can be added to feed. The residence time is short and the cooling rate is fast which results in low degree of thermal degradation, but the outlet temperature is generally 50–70 °C, requiring thermoprotective carriers. The high glass transition temperature (Tg ≈ 115 °C) and the formation of a rigid glassy matrix that immobilizes phage particles (70) make trehalose an excellent protective excipient during spray-drying, particularly for phages. It has been shown that trehalose is an excellent drying excipient for phages, with no measurable loss in phage titer over a variety of drying temperatures when trehalose is the sole excipient.

The application of Design of Experiments (DoE) techniques has allowed for optimization of the spray drying parameters in a systematic way. Tabare et al. (71) tested the viability of the phages of *Pseudomonas aeruginosa* LUZ19 (a podovirus) and 14–1 (a myovirus) at varying inlet temperatures, spray gas flow rates and trehalose/L-isoleucine ratios. They found that inlet temperature did not affect the activity of the phages and that the flow rate of the spray gas had a positive correlation with the viability of the bacteriophages. The results showed that the impact of L-isoleucine on LUZ19 was positive, while the impact on 14–1 was negative, indicating that the impact of excipients on phages is different (71).

In oral delivery applications, pH-responsive polymers like the Eudragit® derivatives have been added to spray dried formulations to protect against gastric degradation. Vinner et al. (50) showed that the viability of *Salmonella* Felix O1 was preserved during spray drying when the phage was formulated with trehalose and Eudragit S100, with no measurable loss in phage titer observed for the formulations with trehalose. Spray drying alone with Eudragit S100, however, led to a reduction of the phage to about 4 log_10_. The reduction in phage activity was at least 2 log_10_ when exposed to the simulated gastric fluid at pH 2 for 2 h, depending on the formulation, which means there is still room for optimization to achieve complete gastric protection (50).

Recently, Tabare et al. (70) developed colon targeted oral delivery systems with excipients of Eudragit® FS30D. The colonic-release polymer was chosen to prevent premature release of the phages in the stomach and small intestine, where the pH is lower. The optimized formulation was able to retain the activity of phage LUZ19 during spray-drying and was resistant to high acidic conditions. The stability studies indicated that the powder was stable for 6 months or more at 5 °C.

Microencapsulation using spray-drying has also been shown to significantly improve phage stability in simulated gastrointestinal conditions. Using a spray dryer, Acar Soykut et al. (72) microencapsulated *Bacillus subtilis* and *Salmonella* phages with 1.33% (w/v) sodium alginate. The maximum titer loss was 2.29 log units for *B. subtilis* phages, 1.71 log units for *S. enteritidis* phages, and 0.60 log units for *S. typhimurium* phages after 2 hours of incubation in simulated stomach juice; free phages lost their viability even after 15 minutes. Similarly, microencapsulation increased phage stability in bile salt medium, with the difference between titers of microencapsulated and free phages reaching up to 3 log units after 3 h of incubation (72).

Suboor, (73) spray-dried a two-phage cocktail (myoviruses SPFM10 and SPFM14) targeting *Salmonella* using formulations containing trehalose, mannitol, leucine, and Eudragit S100. The phages demonstrated high survivability when formulations containing combinations of sugar, amino acid, and polymer were used. When administered to piglets, a decline in *Salmonella* colonies was observed (73).

However, feed mills operate under variable steam pressures and conditioning times; batch-to-batch variability remains a major manufacturing challenge. Furthermore, spray-dried powders often exhibit rapid phage release at gastric pH unless additional enteric coating is applied, which increases cost and complexity. The discrepancy between *in vitro* protection and *in vivo* efficacy highlights the need for dynamic dissolution models that simulate gastrointestinal conditions and the fed-state gastric milieu.

#### 3.2.3 Lipid-based nanoparticles and liposomes

Liposomes and solid lipid nanoparticles (SLNs) offer a versatile platform for phage encapsulation, protecting phages from both acid and enzymes, and enabling surface functionalization for targeted delivery. Lipid-based nanocarriers have been shown to improve phage protection under harsh physiological conditions, enhancing stability, in-vivo retention, and targeted delivery (74).

Liposome encapsulation provides several advantages for phage therapy. It can help treat intracellular diseases like *Mycobacterium* TB, *Listeria* species, *Salmonella* species, and Staphylococcus species by facilitating phage access into phagocytic cells (75). For gastrointestinal infections, liposomes can address the common problem of short residence time of orally ingested phages by providing mucoadhesion. Furthermore, nanoencapsulation in liposomes can overcome poor phage stability under the acidic pH of the stomach (75).

Studies have demonstrated that liposome encapsulation enhances phage efficacy *in vivo*. For instance, chickens infected with *Salmonella* spp. that received treatment with a phage cocktail encapsulated in liposomes demonstrated enhanced effectiveness and extended protection. After 72 h of administration, encapsulated phages were detected in 38.1% of treated animals versus only 9.5% in animals receiving free phages (75). According to Pardo-Freire and Domingo-Calap (75), liposome encapsulation promoted adherence to the intestinal membrane and prolonged presence in the stomach. Similarly, the concentration of phage UAB_Phi20 in the stomach of mice was significantly higher when administered encapsulated, even 6 h after administration, with no decrease in phage titer over that time.

Liposome encapsulation can also protect phages from antibody-mediated neutralization. Singla et al. (76) demonstrated that entrapment of phage in liposomes provided 100% protection from neutralizing antibodies, while un-entrapped phages were neutralized within 3 h of interaction with antibody. Furthermore, while free phages were unable to enter macrophages, liposomes successfully delivered entrapped phages inside macrophages, achieving 94.6% killing of intracellular *Klebsiella pneumoniae* (76). Quantitative data from this study showed that liposome-entrapped phages-maintained titers of 5.18 log PFU/mL after 3 h in the presence of antibodies, whereas free phages were completely inactivated within the same timeframe (76). Chadha et al. (71) similarly reported that liposome-encapsulated phages were protected against neutralizing antibodies compared to free phages, with an encapsulation efficiency of 79.2 ± 5.6% in phage-entrapped liposomes. The liposomal preparation protected mice from death even when treatment was delayed for 24 h, demonstrating the potential of liposome encapsulation to overcome pharmacological barriers to phage therapy (71).

Niosomes, non-ionic surfactant-based vesicles, have also been explored for phage delivery. A study that used transfersomes, nanostructured lipid-based carriers, as transdermal delivery vehicles to encapsulate a cocktail of methicillin-resistant *Staphylococcus aureus* (MRSA) phages showed more stability and persistence than free phages. Transfersome-entrapped phage cocktail-treated rats recovered from experimental thigh infections in 7 days as opposed to 20 days for untreated rats. The transfersome-entrapped phage cocktail protected all test animals with no deaths, even when administered with a delay of 12 h post-infection, unlike free phages (77).

However, high-pressure homogenization or sonication used during SLN and niosome production can shear phage tails, and the high cost of pharmaceutical-grade lipids limits economic viability for food-animal applications (74, 78). PEGylated liposomes have been explored to prolong circulation time by protecting against clearance by the reticuloendothelial system, but rapid opsonization remains a concern after repeated dosing (75, 79).

To improve intestinal targeting, lipid nanoparticles have been surface-decorated with ligands such as lectins that bind to intestinal M cells, facilitating transcytosis. The small size of liposomes is suitable for cellular uptake, and the presence of cholesterol and PEG can protect against clearing by the reticuloendothelial system, improving circulation time (75). While promising, the immunogenicity of surface modifications and their potential effects on gut homeostasis must be carefully evaluated in target livestock species before field application.

#### 3.2.4 Advanced biomaterials (hydrogels, lipid vesicles, polymeric matrices)

Beyond simple capsules, stimuli-responsive hydrogels are being engineered to release phages in response to environmental triggers such as pH changes or bacterial presence. For veterinary applications, injectable thermosensitive hydrogels have been developed for topical and localized phage delivery.

Pluronic® F-127 (Poloxamer 407) is one of the most studied thermosensitive gelling agents for phage delivery (80). Mukhopadhyay et al. (80) developed thermosensitive hydrogels containing varying amounts of PF-127 (15–20% w/w) modified with hydroxypropyl methylcellulose (HPMC) for delivering a phage–antibiotic combination against *Acinetobacter baumannii* wound infections. Most hydrogel formulations had a gelation temperature around skin temperature, making them suitable for topical application. The solidified gels released encapsulated phages and colistin in a sustained manner, achieving a 5.66 log reduction in planktonic bacteria and a 3-log reduction in biofilms. The formulation demonstrated good storage stability with negligible activity loss after 9 months at 4 °C. In an *ex vivo* porcine skin wound infection model, the hydrogel formulation significantly reduced bacterial burden by 4.65 log (80).

For oral delivery, pH-responsive hydrogel microspheres have been designed to protect phages from gastric conditions and release them in the intestine. Yang et al. (81) used electrohydrodynamic spraying to create double-responsive hydrogel microspheres (HMs) made of sodium alginate, hyaluronic acid, and Eudragit S100. For a mixture of *Salmonella* phages, these microspheres had an encapsulation effectiveness of 90%. The formulation allowed for responsive payload release in the colon, extended intestinal retention, and greatly shielded phages from stomach conditions. HMs-encapsulated phages decreased intestine STm burden by about 2000 times and proinflammatory cytokines (TNF-α, IL-6, and IL-1β) to 60% of those in the infected group in a mouse model of colitis caused by *Salmonella Typhimurium*. Interestingly, the targeted phage administration avoided antibiotic-associated microbiota dysbiosis and diarrhea by specifically targeting STm and achieving antibacterial activity comparable to ciprofloxacin (81).

Hydrogels have also been investigated as phage-eluting wound dressings in livestock models. A study using Gum Karaya polysaccharide-based hydrogels supplemented with *Pseudomonas aeruginosa* phages (Pbunavirus MB501 and Phikmvvirus LUZ19) in a porcine deep wound infection model demonstrated a significant synergistic effect. In treated pigs, bacterial load decreased from 7.8 ± 0.4 log CFU/g to 5.1 ± 0.8 log CFU/g of tissue, representing a 2.7 log reduction, compared to a 1.0 log reduction in untreated controls. No adverse effects of the GK hydrogels or phage preparations were observed during the study (82).

Similarly, in a porcine deep wound infection model, an injectable Gum Karaya hydrogel formulation containing Staphylococcus phage 812 K1/420 (Kayvirus genus) was tested against methicillin-resistant *Staphylococcus aureus* (MRSA). Within a week, the bacterial load was significantly reduced (2.5 log CFU/g of tissue) as a result of the combined treatment’s synergistic effect. By day eight, there was a noticeable decrease in the local signs of inflammation, along with wound contraction and re-epithelialization (82).

Carrageenan-based hydrogels have also been developed as phage delivery systems. Narayanan et al. (83) fabricated hydrogel wound dressings using the marine polysaccharide carrageenan to immobilize lytic coliphage vB_Eco2571-YU1 against pathogenic *E. coli*. The immobilized phage showed a sharp decline in bacterial growth during the log phase and, importantly, did not allow for the acquisition of phage resistance by the *E. coli* strain, unlike unimmobilized phages which developed resistance within 9 h. The hydrogels were found to be hemocompatible using a hemolysis assay (83). The comparative advantages, limitations, and representative *in vivo* data for each formulation technology are summarized in Table 2.

**Table 2:** Comparative overview of formulation technologies for phage cocktails in veterinary medicine.

Formulation type	Core materials	Key advantages	Key limitations	Stability in SGF (pH 2, 2 h)	Intestinal release	Representative *in vivo* data	References
Liquid suspension	Tris/NaCl buffer, SM buffer, 2–5% glycerol/sucrose	Low cost, easy preparation, high initial titer; SM buffer preserves infectivity for 30 days at 4 °C	No gastric protection; poor thermal stability; not feasible for large-scale water medication	<1% survival	N/A	Reduced diarrhea incidence in piglets; 56.67% survival in zebrafish model	(54, 56, 146)
Lyophilized cake	Trehalose + mannitol/skim milk; sucrose + gelatin; whey protein/trehalose	Excellent long-term storage (up to 10 months at 25 °C); sucrose/gelatin maintains viability for 10 months; cold-chain optional	Hygroscopic; no gastric protection; reconstitution needed; requires desiccants	<5% survival without enteric coating	N/A	2.0 log_10_ *Campylobacter* reduction in broilers; 56.67% survival in zebrafish embryo model at MOI 0.1	(51, 146)
Alginate microspheres	Ca-alginate; alginate–pectin (3:1); alginate–carrageenan + CaCO_3_	pH-responsive enteric release; mild gelation; biocompatible; ALG-CG-CA shows higher phage recovery in acid	Porous to pepsin; premature dissolution by feed phosphates; rumen microbes degrade alginate	50–80% (alginate alone); 85% with alginate–pectin; high recovery with ALG-CG-CA	60–80% release in SIF; complete release within 6 h at pH 6.8	1.2 log_10_ ileal ETEC reduction in pigs (vs. 2.8 log for direct instillation)	(35, 68, 69)
Alginate–chitosan complex	Ca-alginate + chitosan coating; chitosan-alginate-CaCl₂	Superior acid/enzyme resistance over alginate; >90% SGF survival; complete bile protection	Complex manufacturing; incomplete *in vivo* release; chitosan requires acetic acid (affects phage survival)	>90% survival (3 h in SGF); encapsulated phage decreased only 0.67 log at pH 2.4	Rapid release (30 min) in SIF; complete release within 6 h at pH 6.8	3.5 log_10_ *S. typhimurium* reduction in simulated pig gut; 2.8 log_10_ faecal reduction in pigs; 3 log *Campylobacter* reduction in broilers	(35, 68, 110)
Spray-dried microcapsules	Trehalose + mannitol + leucine (60:20:20); whey protein + trehalose; trehalose-l-leucine; lactose-leucine	Scalable; high-throughput; survives pelleting (70–75°C); lactose provides 0.3–0.4 log loss (better than trehalose 0.5–0.9 log); FPF > 50% for inhalation	Outlet temperature stress (50–70 °C); often needs enteric coating; rapid gastric release; leucine alone is poor stabilizer	Poor without coating; depends on excipient composition	Fast release at gastric pH unless enteric-coated	55% PWD reduction, 40% mortality reduction (1,200 pigs); 3.8 log_10_ *Salmonella* reduction in broilers; 90% phages survived 2 h in SGF	(147–149)
Liposomes/SLNs	Cationic liposomes (phospholipids, cholesterol); DSPC/cholesterol/Tween 80/DOTAP	100% protection from neutralizing antibodies; enables macrophage entry (94.6% killing of intracellular bacteria); synergistic biofilm eradication with antibiotics	Shear damage during production; costly lipids; immunogenicity (PEGylated); overestimation of encapsulation yield for tailed phages	High protection (3-fold over free phages in bile)	Controlled release	94.6% killing of intracellular *K. pneumoniae* in macrophages; liposome-entrapped phage constant titer after 3 h antibody exposure; synergistic biofilm eradication with amikacin	(75, 76, 145)
*In situ*-forming hydrogels	Pluronic F-127 + HPMC (14% + 1.5%); Poloxamer 407 (15–20% w/w) + HPMC	Sustained release (24–72 h); thermosensitive (gels at 33 °C); 30s gelling time; 10 × viscosity increase upon gelation; easy intramammary instillation	High cost for veterinary market; limited oral applications; complex manufacturing	Not applicable (local delivery)	Not applicable	Improved rabbit keratitis outcomes; 90% bacteriological cure in subclinical *S. aureus* mastitis (cows); 78% cure in clinical mastitis	(80, 150, 151)

## 4 Delivery routes and biopharmaceutical performance

A critical determinant of phage therapy success is the alignment between the chosen delivery route and the formulation’s protective features.

### 4.1 Oral delivery (gavage, feed, drinking water) – requirements for gastric protection

Oral administration is the most practical route for mass medication in poultry and swine. Gavage is used to obtain accurate doses in experimental situations but not on farms. Feed and water ad libitum delivery are standard and thus phages are subjected to the highest physicochemical stresses, such as gastric acid, pepsin, bile, intestinal enzymes and, in the case of feed, thermal pelleting. Therefore, gastric acid protection (enteric coating/encapsulation) and water stability for 24–48 h at ambient temperature is required for oral formulations (12).

Farm drinking water quality is frequently neglected. Residual chlorine disinfection can kill phages and organic material in water troughs can bind the phages, thus decreasing the actual dose of the animal. Thus, water-stable excipients, like sugars and salts, are essential for the stability of phages in medicated water lines. Kim et al. (56) formulated a stabilizing solution using sugars and salts, which was able to stabilize the phages for up to 48 weeks in distilled water at room temperature.

Despite impressive *in vitro* survival data, oral phage cocktails often achieve only modest intestinal colonization and variable pathogen reductions *in vivo*. This difference can be explained by several reasons: the first is that in young animals, the transit time through the gastrointestinal tract is short, which decreases the time for the phage to be released from the capsule; the second is that the volume of fluid in the large intestine is high, which would cause the phage to be diluted; the third is that the commensal microbiome which inhabits the gut would compete for receptor sites or produce inhibitory metabolites; and the fourth is that the local concentration of the phage would not be sufficient to cross the replication threshold (inundation threshold) in the entire gastrointestinal tract (84, 85). Fujimoto’s (86) study of the significance of phage therapy for controlling intestine commensal infections has revealed that the intestinal system is a special anaerobic environment in which phages are highly specific with regard to their host bacteria.

In a retrospective observational study of 100 consecutive cases of personalized phage therapy, Pirnay et al. (84) showed that phage resistance and phage immune neutralization were documented in patients, with 38.5% of patients screened for phage immune neutralization. Moreover, the study showed that 77.2 and 61.3% of infections, respectively, had clinical improvement and elimination of the targeted bacteria, indicating that phage therapy could be used in combination with antibiotics to achieve positive results.

The dose-volume relationship is also important; when phages are administered in a small volume of concentrated slurry, their survival in the stomach is greater than when administered in large volume drinking water due to the lower surface area to volume ratio and faster gastric emptying of liquids, allowing for less time to be exposed to the stomach’s acid.

### 4.2 Parenteral administration (IM, SC) – sterility and systemic distribution

Intramuscular (IM) or subcutaneous (SC) injection bypasses gastrointestinal barriers and is suitable for systemic infections or infections at confined sites (e.g., respiratory infections in cattle, septicemia in poultry). The sterilization and apyrogenicity of the phage preparation is of utmost importance, and the endotoxin removal from phage lysates should be validated to below regulatory limits such as <5 EU/kg body weight for intravenous applications (87).

A number of methods for eliminating endotoxins from phage preparations have been reported. In their investigation of polyethylene glycol (PEG)-NaCl precipitation as a scalable technique for endotoxin separation from phage M13, Branston et al. (88) showed that supplementing with up to 2% (v/v) Triton X-100 resulted in a 5.7 log_10_ reduction in endotoxin concentration over three rounds of precipitation while retaining more than 97% of the phage. When it comes to removing endotoxins from protein solutions, this approach is comparable to the well-known Triton X-114 aqueous two-phase system methodology (88).

Van Belleghem et al. (87) conducted a thorough investigation that examined seven distinct endotoxin removal techniques for five phages (one *Staphylococcus aureus* phage and four *Pseudomonas aeruginosa* phages). They showed that the strongest reduction, with an endotoxin removal effectiveness of up to 99%, was achieved by CsCl density purification at an initial concentration of 10^12^–10^13^ PFU/mL. Other purification techniques produced varying efficacy (23–99%) and occasionally caused significant phage titer reductions (87).

In a retrospective observational analysis of 100 consecutive cases of individualized phage therapy, Pirnay et al. (84) found that 38.5% of screened patients had phage immune neutralization, and 43.8% of reviewed patients had *in vivo* selection of phage resistance. In 77.2 and 61.3% of infections, respectively, there was clinical improvement and eradication of the targeted bacteria; the likelihood of eradication was 70% lower in the absence of concurrent antibiotics.

While parenteral delivery ensures that 100% of the dose reaches systemic circulation initially, rapid clearance by the liver and spleen limits the plasma half-life. Merril et al. (45) showed that the ability of mammalian host defense systems, specifically the reticuloendothelial system’s organs, to eliminate phage particles from the circulatory system hindered the therapeutic use of phages. They created a serial-passage method in mice to select for phage mutants that could stay in the circulatory system for longer in order to solve this. Using this method, they were able to isolate long-circulating mutants of *Salmonella* phage P22 and *Escherichia coli* phage lambda, showing that these mutants were more effective as antibacterial agents than the corresponding original strain in bacteremic mice. The relevant mutation changed the primary phage head protein E, according to a comparison of the parental and mutant lambda capsid proteins (45).

More recent studies have confirmed rapid clearance kinetics in large animal models. In a sheep model of fracture-related infection, Peez et al. (89) assessed phage pharmacokinetics and found that phages were quickly removed from circulation following intravenous delivery, with no phage remaining after 240 min. Phage neutralization accelerated in infected hosts, reaching maximum levels by day 13 compared to day 50 in non-infected animals, and increased during therapy, peaking at 99.9% by the end of treatment.

Moreover, repeated parenteral administration induces phage-neutralizing antibodies (IgG and IgM), which can abrogate therapeutic efficacy upon subsequent dosing. A study on intranasal versus intraperitoneal phage delivery for *Pseudomonas aeruginosa* lung infections demonstrated that intraperitoneal administration triggered significant IgG, IgM, and IgA production and resulted in decreased survival, whereas intranasal delivery elicited undetectable antibody responses and significantly improved survival (90). This highlights that the route of administration profoundly influences the development of antibody-mediated neutralization, with localized delivery routes potentially overcoming this limitation.

In poultry, in ovo injection of phages has shown promise for controlling bacterial infections. A 2025 study evaluating *Salmonella* phage administration via the in ovo route in broiler chickens reported that phage-inoculated eggs had a hatching rate of 96.97%, with no significant differences compared to non-inoculated controls. Phage detection rates in cecum samples ranged from 57.14 to 87.50% depending on the inoculation site, with average phage titers of 3.66 to 4.98 log_10_ PFU/sample (91).

Localized injection at the infection site can achieve high tissue concentrations while reducing systemic exposure. A study by Cheetham et al. (92) on *Dichelobacter nodosus*, the causative agent of ovine footrot, identified the bacteriophage DinoHI and its interactions with integrated genetic elements that modulate virulence, providing a foundation for phage therapy development against this pathogen.

### 4.3 Intramammary infusion – localized delivery for mastitis

Bovine mastitis, predominantly caused by *S. aureus*, *Streptococcus uberis*, and *E. coli*, is a major target for phage therapy. Intramammary infusion deposits phages directly into the mammary gland cistern, achieving high local concentrations while minimizing systemic exposure (93).

The milk environment presents unique challenges for phage therapy. Milk is a complex medium composed of lipids, milk proteins (caseins, whey proteins), and an endogenous bacterial flora. Previous studies have demonstrated that heat treatment (UHT > pasteurized) and skimming of milk have a positive effect on the bactericidal activity of phages, suggesting an inhibition of phage binding ability to host cells in raw milk, mainly attributed to lipids and whey proteins (94). Titze and Krömker (94) investigated the bactericidal activity of obligately lytic phages against *S. aureus* in pasteurized and raw milk, demonstrating that phage efficacy is significantly influenced by the milk matrix.

The efficacy of phage therapy for intramammary infections has been demonstrated in several model systems. Local intramammary treatment is tolerated and may enhance bacteriological cure compared to non-treatment, according to randomized, negative-controlled pilot research assessing a *S. aureus* bacteriophage cocktail given locally to the mammary gland in calves. The study concluded that intramammary treatment with bacteriophages has great potential as it is safe and free of antimicrobial residues (93).

When vB_EcoM-UFV13 was administered intramammary against mammary pathogenic *E. coli* (MPEC) in a murine mastitis model, the bacterial burden was reduced 10-fold. Following viral therapy, pro-inflammatory cytokines such TNF-α and IL-6 were found, suggesting immunological activation (95).

For *Streptococcus uberis* mastitis, studies have characterized phages with lytic activity against clinical isolates. Phages CP1 and CP2 each reduced *S. uberis* counts by 86 and 83%, respectively, in lethal dose assays. Phage replication cycles were observed to take between 50 and 60 min (96).

Engineered endolysins have also shown promise for bovine mastitis treatment. A top candidate endolysin developed against streptococcal mastitis pathogens demonstrated a 4.0 log killing efficacy against *S. uberis*, with similar activity against *S. agalactiae* and *S. dysgalactiae*, and showed intracellular activity in bovine mammary epithelial cell lines (97).

### 4.4 Aerosol/spray – respiratory delivery

For respiratory pathogens such as *Actinobacillus pleuropneumoniae* in swine and *Mannheimia haemolytica* in cattle, aerosolized phage delivery can target the lung mucosa directly. Phage aerosols must be formulated to withstand the shear forces of nebulization and desiccation in the air stream.

The choice of nebulizer type significantly impacts phage viability during aerosolization. A comprehensive study by Cao et al. (98) demonstrated that liposome-encapsulated phages preserved viability during nebulization, with titer losses below 0.4 log_10_ for encapsulated phages, while non-encapsulated phages experienced titer reductions of up to 1.23 log_10_, especially during jet nebulization. Vibrating mesh nebulization achieved higher phage recovery (>90%) and respirable fractions (>70%), which are desirable for lung deposition (98).

Astudillo et al. (99) evaluated the structural effects of three distinct nebulizer types—air-jet, vibrating-mesh, and static-mesh—on the structural stability of a *Myoviridae* phage (PEV44) active against *Pseudomonas aeruginosa* using transmission electron microscopy. Following nebulization, the percentage of “broken” phages (capsid split from tail) increased dramatically; the air-jet nebulizer caused more damage (83% broken) than mesh-type nebulizers (50–60%) (99). Phage shape was found to be a crucial component, with survival during aerosolization being determined by the integrity of the capsid and tail. For larger, tailed phages, jet nebulizers were shown to preserve a higher proportion of viable phages, whereas vibrating mesh nebulizers more effectively delivered tailless phages (100).

Nebulization has been demonstrated to be protected by encapsulation strategies. The encapsulation efficiency of short-tail phages and long-tail phages was 77.9 and 89.9%, respectively, after lipid-based encapsulation with DSPC, cholesterol, Tween 80 and DOTAP. The inactivation of phages was significant for unbound phages and modest for encapsulated phages (<0.4 log_10_) (98).

Formulations of phage powder for lung delivery have been developed. An additional study involved the addition of Phage vB_AbaM-IME-AB406 to dry powders of trehalose, mannitol, and L-leucine, demonstrating that mannitol increased the dispersibility of the phage and provided storage stability with less than one log titer loss after 6 months at high excipient concentration (101).

In poultry, a trial was conducted to assess the efficacy of aerosol spray of bacteriophage to reduce mortality of broiler chickens due to *E. coli* respiratory infection and it was found that aerosol spray of phage can significantly reduce mortality. Aerosol phage treatment was found to be effective in a severe infection model, with 10^4^ CFU being injected into the thoracic air sac, where mortality was reduced from 50 to 20% upon treatment immediately after challenge. Phage have also been shown to be effective at reducing mortality when administered immediately after challenge, with a 53 to 17% reduction in deaths (102, 103) after intramuscular injection. The relative inefficiency of spray treatment as compared to intramuscular injection, however, was attributed to the failure to achieve high levels of phage in the blood using the spray delivery system (103).

Large animal models of respiratory pathogens have been developed. The aerosol challenge model for *M. haemolytica* pneumonia in colostrum-deprived calves was validated, and consistently induced bronchopneumonia with typical lesions after aerosol challenge with 1 × 10^10^ CFU (104).

There is potential for aerosol delivery of phage for livestock respiratory infections, as it has been shown to be technically possible for phage to survive during nebulization and could be used for prophylactic and therapeutic purposes (98). There are still issues to be addressed, such as structural damage to phages during nebulisation, swift clearance from the respiratory tract, and the need for optimized formulations and delivery devices specific to the morphology of the phages (100).

### 4.5 Comparative analysis of delivery vs. formulation requirements

A comparative synthesis shows that there is no universal formulation, each delivery route requires specific protection. Enteric-coated and thermostable products are required for oral formulations, products that are ultrapure and endotoxin low are needed for injectables, products with bioadhesive sustained release properties are beneficial for intramammary formulations, and products that do not desiccate or shear are required for aerosols. One major error in the early translational research was the use of a single “stable” liquid formulation for all routes, resulting in different efficacy reports. It highlights the importance of considering on-farm delivery logistics in the development of pharmaceuticals. The different production routes of bacteriophage cocktails in various animal species are shown in Figure 3.

**Figure 3:**
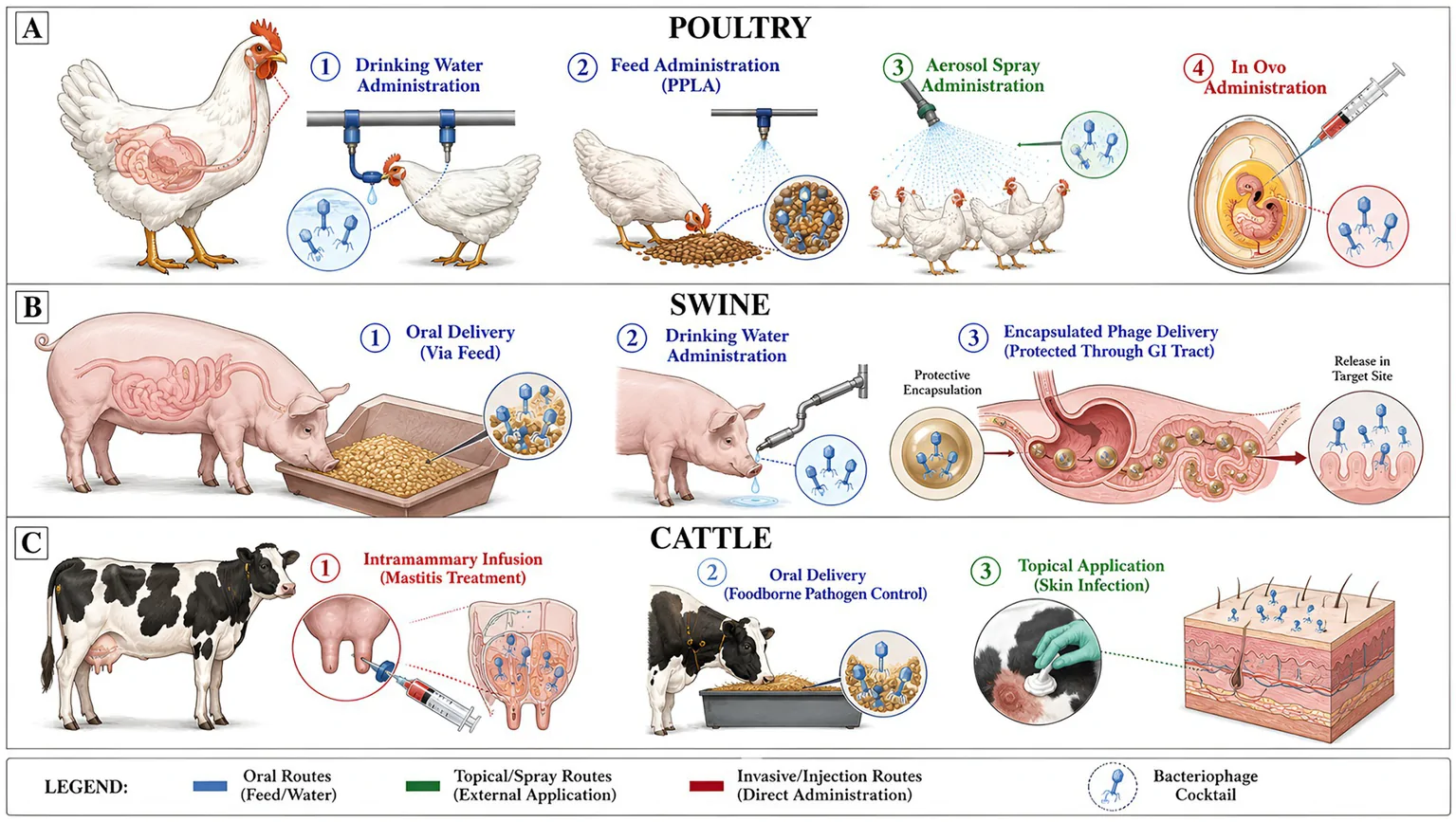
Schematic representation of bacteriophage cocktail delivery routes in production animals. The figure is divided into three panels corresponding to the major livestock species: **(A)** Poultry, showing drinking water administration, feed administration via post-pellet liquid application (PPLA), aerosol spray delivery, and in ovo administration; **(B)** Swine, showing oral delivery via feed, drinking water administration, and encapsulated phage delivery with gastric protection; and **(C)** Cattle, showing intramammary infusion for mastitis treatment, oral delivery for foodborne pathogen control, and topical application for skin infections. Color coding indicates route type: blue arrows denote oral/enteric routes, green arrows denote spray/topical routes, and red arrows denote invasive/injection routes.

The regulatory pathways are route specific and greatly impact development timelines and costs. The sterility and endotoxin specifications for parenteral products are more stringent than those for aerosols used topically or intramammary infusions (105). Veterinary phage therapies are regulated in the EU by Regulation (EU) 2019/6, and the EMA has developed guidelines on quality, safety and efficacy (105). Phage products can be under FDA (therapeutic) or USDA (food safety) control in the USA (105). The regulatory harmonization of phages remains a key challenge, as they might be classified as veterinary medical products, magistral preparations, food additives, or biocides, none of which were designed for phage-based products (11). Flexibility is provided by the EMA and EDQM but there are some concerns regarding the manufacturing feasibility at commercially viable costs (11, 105).

There are few and still needed cost–benefit analyses. At the poultry level, an analysis of the cost of AI-guided phage therapy in broiler chickens estimated the cost at 2 × to 10 × the antibiotic baseline, ranging from $0.10 to $0.50 per bird, compared to $0.05 per bird for antibiotics (106). These extra costs have to be compensated by improvements in productivity in terms of reduction in mortality (0.2–3%), feed conversion ratio (0.2–3%) and weight gain (0.1–2%) (106). The cost-effectiveness of phage therapy has yet to be proven in aquaculture.

The need of the hour is for a unified regulatory framework that can accommodate the unique biological characteristics of phages in different delivery modes, to speed up commercial translation (84). As Pirnay et al. (84) pointed out, frameworks should enable defined phage products and personalized strategies, both alone and in combination with other antimicrobials.

## 5 Therapeutic outcomes in major production species

### 5.1 Poultry (broilers and layers)

Poultry, particularly broilers, have been the most extensively studied species for oral phage therapy due to the economic importance of *Salmonella* and *Campylobacter* control and the feasibility of water/feed medication.

A systematic review and meta-analysis of 20 studies that investigated the effect of phage on the reduction of *Salmonella* spp. in poultry meat, revealed an overall *Salmonella* spp. reduction rate of 1.729 log CFU/cm^2^ (95% CI: 1.548–1.910) (107). The study revealed that the activity of phages increases with time and the concentration of *Salmonella* decreases with an increasing dose of bacteria and phages. It was discovered that phage therapy worked better against *S. enteritidis* than against other species (107).

Phage efficacy has been greatly enhanced by the use of encapsulation. The antibacterial activity of chitosan/alginate films loaded with *Salmonella* phages was successfully confirmed when the films were stacked layer-by-layer, which resulted in the reduction of *Salmonella* counts on contaminated chicken meat at pH 7.0 (108). The ability of alginate to increase gastrointestinal stability and bactericidal activity of oral *Salmonella* phage cocktails in poultry feed has been demonstrated, and a recent study confirmed that alginate can improve the survival of phages in the digestive tract of poultry (109).

A lot of research has been done on phage therapy for *Campylobacter*. Wagenaar et al. (110) demonstrated that phage therapy in broilers resulted in a significant reduction in the counts of *C. jejuni* at the initial stage followed by a stabilization of the bacterial counts after 5 days at a level 1 log lower than the control group. The preventive groups had a delay in colonization, and a first log reduction, according to Wagenaar et al. (110).

According to a more recent study by Peh et al. (111), a phage cocktail containing group II and III phages dramatically decreased fecal *Campylobacter* excretion and *Campylobacter* concentrations in the colon of broilers. Using a targeted development method, the study refined phage cocktails, demonstrating that mixing phages leads to greatly increased lytic activity. This was effectively translated into practical application in settings similar to field conditions (111).

The emergence of phage resistance remains a challenge. Scott et al. demonstrated that in broiler chickens treated with lytic bacteriophages, *Campylobacter* isolates surviving phage predation exhibited genomic rearrangements, including intra-genomic inversions of up to 590 kb (one-third of the genome) (112). These rearrangements conferred three phenotypes: resistance to virulent bacteriophage infection, inefficient colonization of the broiler chicken intestine, and production of infectious bacteriophage CampMu (112). Critically, the inability to replicate bacteriophage was associated with a significant fitness cost that rendered resistant strains competitive only in the presence of bacteriophage (113).

Bacteriophage supplementation has been associated with improved growth performance in broilers. Studies have reported that bacteriophage supplementation enhances growth performance by increasing body weight gain, improving feed efficiency, and positively modulating gut microbiota by killing targeted pathogens while promoting beneficial commensal bacteria. The narrow host range of most phages minimizes off-target effects on commensal bacteria, though cocktails containing broad-host-range phages warrant further investigation.

### 5.2 Swine

In swine, the primary targets for phage therapy are enterotoxigenic *E. coli* (ETEC) causing post-weaning diarrhea (PWD) and *Salmonella Typhimurium*.

A meta-analysis of 11 challenge studies involving 198 piglets quantified the effects of bacteriophage therapy on bacterial load. Prophylactic dosing produced a moderate and highly consistent reduction (SMD = −0.64, *p* < 0.001; I^2^ = 19.41%), whereas therapeutic dosing produced a larger but more variable effect (SMD = −1.29, *p* < 0.001; I^2^ = 66.77%). Reductions were significant for both *E. coli* (SMD = −0.79, *p* = 0.014) and *Salmonella* (SMD = −0.90, *p* < 0.001) models. Encapsulated phages produced larger reductions than non-encapsulated formulations (SMD = −1.51, *p* = 0.026 vs. SMD = −0.70, *p* < 0.001), although the moderator test was not significant. In-feed delivery yielded more consistent outcomes than oral gavage (I^2^ = 21.98% vs. 63.91%) (114).

An updated review by Chen et al. (115) systematically examined bacteriophage applications in weaned piglets, confirming that phages can provide practical guidance for controlling diarrhea and intestinal health problems. The review highlighted that several studies have demonstrated the efficacy of phages in mitigating pathogen phenotypes in piglets, including ETEC-specific bacteriophage reducing diarrhea percentage.

Microencapsulation has been shown to improve phage stability and delivery in swine. Dlamini et al. (116) demonstrated that alginate–carrageenan encapsulation with 1% (w/v) CaCO_3_ significantly improved phage viability at pH 2–2.5, reducing phage loss to <0.5 log_10_ PFU/mL even after 1 h at pH 2. Phages were efficiently released from microcapsules following a shift to neutral pH (7.5), simulating passage to the duodenum.

Ten phages from the *Siphoviridae* and *Myoviridae* families were used to create an anti-*Salmonella* phage cocktail. Microencapsulation using sodium-alginate-based techniques reduced the cocktail titer by just 1.0–1.5 logs (premicroencapsulation: 10.4 log_10_ PFU/mL; postmicroencapsulation method one: 9.2 log_10_ PFU/mL; postmicroencapsulation method two: 8.9 log_10_ PFU/mL). For up to 14 days, microencapsulated phages did not significantly decrease in titer at either 4 °C or 22 °C (117).

Bacteriophages may be a viable substitute for antibiotics and might be used to enhance pig growth performance, according to an *in vivo* evaluation of bacteriophages specific to multidrug-resistant *E. coli* on fecal bacterial counts and microbiota in nursery pigs. *Salmonella* colonization in pigs has been demonstrated to be decreased by direct feeding of microencapsulated bacteriophages (118).

The long-term evolutionary consequences of phage therapy in swine have been examined. Research on bacteriophage resistance mechanisms has identified that resistant mutants may carry mutations in lipopolysaccharide (LPS) core biosynthetic genes such as *waaG* and *waaQ* (119). These mutants are often outcompeted by wild-type strains in the absence of phages, indicating a significant fitness cost that might be exploited therapeutically.

### 5.3 Ruminants (bovine: dairy and beef)

Phage therapy in adult ruminants faces the formidable barrier of the forestomach—rumen, reticulum, and omasum—whose microbial density and complex biochemistry can rapidly inactivate or outcompete introduced phages.

Early attempts to orally deliver phage cocktails against *E. coli* O157: H7 in cattle met with limited success. Bach et al. (120) showed that although three lytic *E. coli* O157 phages (rV5, wV8, and wV11) could survive in ruminal fluid from cattle fed forage and concentrate for a full day, there was no discernible lytic activity against *E. coli* O157: H7 in ruminal batch cultures, and none of the phages changed fermentation parameters. *In vivo* studies have similarly reported that phages could be recovered in the rumen but declined over time (approximately 92 h), with shedding varying between animals (121).

The rumen presents unique challenges for phage therapy. Research on alginate-chitosan microbeads and thermos-responsive hydrogels for phage delivery has demonstrated that release kinetics from biomaterials are impacted by both the biomaterial and the phages themselves (122). Studies have shown potential for local delivery of phages using hydrogels, with results indicating that phage release kinetics can be modulated for both rapid burst release and controlled, extended release (122).

Local administration has shown far greater success in ruminants. A randomized, negative-controlled pilot study evaluating an intramammary *S. aureus* bacteriophage cocktail in dairy cows reported that local intramammary treatment was well-tolerated and may improve bacteriological cure compared to non-treatment (93). Due to their safety and lack of antibiotic residues, the use of bacteriophages to treat intramammary infections has enormous potential (93). 23 udder quarters infected with *S. aureus* were randomly assigned to a treatment group (*n* = 16) and a control group (*n* = 7) in a later effectiveness study conducted on a different farm. Five intramammary infusions of the phage mixture were administered to the treatment group at intervals of 12 hours. As a result, the treatment group’s bacteriological cure rate was 81.3% (13/16), while the control group’s was 28.6% (2/7) (*p* = 0.026) (93).

In 24 clinical instances of cattle with staphylococcal mastitis, Gautam et al. (123) compared the therapeutic efficiency of intramammary bacteriophage with antibiotic therapy. Four treatment groups were randomly assigned to the animals: simultaneous therapy (T4), antibiotic alone (T1), bacteriophage followed by antibiotic 8 hours later (T2), and antibiotic followed by bacteriophage 8 hours later (T3). The sequential bacteriophage then antibiotic group (T2) had the highest recovery rate (83.33% (5/6)), followed by T4 and T3, while antibiotic alone (T1) had the lowest efficacy (123). This method may aid in lowering antibiotic resistance in dairy cattle and provides a viable alternative for the treatment of staphylococcal mastitis (123).

The lytic effectiveness of bacteriophages against *S. aureus* isolates from bovine mastitis was examined by Titze et al. (124). Three phages (STA1. ST29, EB1. ST11, and EB1. ST27) make up the phage mixture, which was able to lower the density of *S. aureus* bacteria in pasteurized milk, and it was still able to do so in raw milk, but to a lesser extent than in pasteurized milk. At least one of the tested phages was able to lyse nearly two thirds of the 92 isolates that were examined (124).

A notable exception to the rumen barrier is the neonatal calf, whose rumen is not yet functional and whose gastric pH (abomasum) is comparable to that of monogastrics. Bacteriophage supplementation in pre-weaned dairy calves has been shown to reduce the duration of neonatal diarrhea. A study of 200 Holstein × Gyr crossbred female calves divided into control (no supplementation) and phage groups (1 g/day from day 3 to day 70 of life) reported that the phage group had fewer days with diarrhea (PHAGE: 4.68 days, CON: 6.61 days; *p* = 0.03) (125). The average daily gain of the phage group was higher up to 80 days of life (*p* < 0.05), and fecal samples of three animals in the phage group (vs. nine in control) were positive for *E. coli* after PCR tests (125).

PhageLab, in partnership with MSD Animal Health, has launched PHAGEIN in Brazil, a cutting-edge biotechnology solution for managing calf health that leverages bacteriophages to selectively target pathogenic bacteria such as *E. coli* and *Salmonella* while preserving beneficial gut microbiota (126). This represents a significant commercial milestone, indicating that oral phage therapy for enteric infections in pre-ruminant calves is a viable, commercially relevant niche (126).

For bovine respiratory disease (BRD), phage-derived endolysins have shown promise. Moqaddes (127) engineered bacteriophage-derived endolysins, either unmodified or fused to polycationic nanopeptides (PCNPs), and evaluated their activity against *Mannheimia haemolytica*. While native endolysins exhibited strong muralytic activity, PCNP-fused endolysins showed greater antibacterial activity, achieving up to 4.5 log_10_ reductions in viable bacteria, particularly with EDTA (127). However, conventional phage isolation and plaque assays using BRD-associated clinical samples failed to recover active lytic phages, suggesting that prophages or extensive antiviral defense systems may restrict phage recovery (127).

The induction of an anti-phage response did not significantly affect antibacterial efficacy, according to a study on anti-phage antibodies in calf sera after treatment of *E. coli* and *M. haemolytica*-specific bacteriophages. Improvements in health status, a protective impact that persisted for at least 3 weeks following the last phage dose, and a notable decrease in pathogenic *E. coli* in feces and *M. haemolytica* in calve’s nasal cavities were among the measurable benefits (128).

### 5.4 Aquaculture (salmon and shrimp)

Aquaculture species present unique delivery challenges because phages are administered in an aqueous environment where dilution is immediate and continuous. In flow-through or recirculating aquaculture systems, phage titers decline rapidly due to water exchange, UV disinfection, and adsorption to particulate matter.

In marine shrimp (*Litopenaeus vannamei*), phage therapy against *Vibrio parahaemolyticus* causing acute hepatopancreatic necrosis disease (AHPND) has been investigated. Bacteriophages can impact bacteria through a variety of processes, such as lowering bacterial concentration through the lytic process or altering genes encoding bacterial pathogenicity, according to a 2024 study on *Vibrio* spp. phages isolated from shrimp farming in the Mekong Delta (129). Phage KG6 decreased bacterial abundance without altering gene sequences, however phages ST9 and ST10 altered nucleotides at four locations in comparison to the *V. parahaemolyticus* sequence (129). If such effects can lessen or negate bacterial pathogenicity, these findings open up new avenues for phage therapy research (129).

Ecology and evolution of *Vibrio* phage interactions have been studied in great detail in natural populations. In recent years, the field of *Vibrio* phage research has seen significant progress, with high-throughput assays and genomic tools providing new insights into the specificity and host range of *Vibrio* phages and the evolutionary dynamics driving these interactions (130). One of the key discoveries is that mobile genetic elements (MGEs) encode bacterial defense systems that influence the coevolution of phages and bacteria. The discovery of such a phage satellite as PICMI highlights the role of MGEs in the transfer of antiviral systems, which has an impact on ecological and evolutionary dynamics (130). The paradox of the generalist *Vibrio* phages, which are rare but have wide hosts, is also discussed (130).

Bernard et al. (131) investigated the genome of a *Vibrio crassostreae* host, and the genomes of the associated Schizotequatroviruses, from an oyster farm. The conserved genomic architecture of these phages, with broad host ranges, is interrupted by recombination hotspots. Importantly, some strains were found to be evolutionarily flexible, with the ability to switch from *ompK* to *lamB* (131). Although the host range is broad, there were few reports of schizotequatroviruses in the environment. This may be because of ecological constraints or fitness costs, including their preference for generalist *Vibrio*s in seawater, or for the patho-phylotypes selected for oyster farms (131). These discoveries lay the groundwork for upcoming phage applications in aquaculture and shed light on the genetic and ecological trade-offs influencing Schizotequatrovirus generalism (131).

Deng et al. (132) analyzed the coexistence strategies of phage-prokaryote in shrimp culture pond ecosystems, and found that the interactions between phages and prokaryotes regulate the diversity of microbial community in the intestine and sediment microhabitats (132). The knowledge of this ecological aspect is essential for the design of efficient phage-based intervention in aquaculture (132).

The genetic structure of the host populations is represented by the *Vibrio* phage interaction networks (133). Environmental *Vibrio* phage research has revealed these interactions are highly modular and are regulated by a specific matching between genus of *Vibrio* phage and clades of *Vibrio*. After adsorption, intracellular defense mechanisms further restrict the number of strains that can be successfully infected and lysed (133).

Various routes of phage therapy have been tested for salmonids. *Aeromonas salmonicida* subspecies *salmonicida* have been isolated from aquaculture settings; studies have been conducted on the polyphasic identification of *A. salmonicida* and virulence characterization (134). A careful analysis of these species-specific results clearly shows that when the formulation and delivery method is carefully tailored to the digestive physiology of the animal and the niche of the pathogen, phage cocktails can provide clinically significant reductions of pathogen load and mortality. On the other hand, failures are often due to the failure to consider the dynamic *in vivo* environment: the stability of the *in vitro* environment is assumed to be the same as that of the intestinal environment, or the *in vitro* encapsulation is assumed to be stable in the aquatic environment. Table 3 summarizes the therapeutic outcomes of phage cocktail applications across major production species, including poultry, swine, ruminants, and aquaculture, with key clinical endpoints and practical considerations.

**Table 3:** Summary of therapeutic phage cocktail outcomes in food-producing animals.

Species	Target pathogen(s)	Delivery route & formulation	Key clinical endpoint (magnitude)	Practical considerations	Reference
Broilers	*Salmonella Enteritidis*	Oral gavage, drinking water, aerosol spray (10^7^ PFU/mL)	Prophylactic: 1.92 log_10_ reduction (Day 3); Drinking water: 1.62 log_10_; Aerosol: 1.12 log_10_ (least effective)	Prophylactic administration most effective; aerosol spray least effective; route of delivery significantly impacts efficacy	(152)
Broilers	*Salmonella* spp.	Phage cocktail in feed/water	1.0–1.4 log_10_ CFU/g reduction in cecal contents; improved weight gain	Treatment improved weight gain; effective for reducing target bacteria colonization	(15)
Piglets	ETEC K88 (F4)	Phage cocktail (oral)	19.2% lower cumulative diarrhea burden (*p* = 0.044); 61.9% higher average daily gain (*p* = 0.065); significant *E. coli* reduction (*p* = 0.009)	Phage therapy shows potential as antibiotic alternative; no major shifts in microbial diversity; phage replication detected in feces	(153)
Piglets	ETEC	Phage cocktail (prophylactic)	Prophylactic dosing produced moderate and highly consistent reduction (SMD = −0.64, *p* < 0.001; I^2^ = 19.41%); encapsulated phages: SMD = −1.51 (*p* = 0.026) vs. non-encapsulated: SMD = −0.70 (p < 0.001)	Prophylactic more consistent than therapeutic; in-feed delivery more consistent than oral gavage; encapsulation improves efficacy	(114)
Dairy Cows	*S. aureus* (mastitis)	Intramammary phage cocktail (5 infusions at 12-h intervals)	81.3% (13/16) bacteriological cure rate vs. 28.6% (2/7) in control group (p = 0.026)	Safe and free of antimicrobial residues; well-tolerated; first negative-controlled *in vivo* study; larger multicenter studies needed	(93)
Beef Cattle	*Salmonella* spp.	Phage cocktail applied to feedlot pen surface	Significant reduction on cattle hides at Days 14 and 21 (combination treatment most effective)	Pre-harvest intervention may reduce *Salmonella* populations; combination with water/manure treatments most effective	(154)
Aquaculture	*Vibrio campbellii*	Phage vB_VcaP_R24D and vB_VcaP_R25D	High stability at 4–45 °C and pH 3–10; robust lytic efficiency during logarithmic growth	Both phages are podovirus morphotypes; short latent period (<10 min); no lysogenic genes or virulence factors; promising biocontrol agents for Vibriosis	(155)

## 6 Challenges and future perspectives

### 6.1 Scale-up and manufacturing costs

The production of high-titer phage lysates at industrial scale (thousands of liters) is technically feasible, as demonstrated by existing commercial phage products for food safety (e.g., ListShield™, PhageGuard). However, a significant obstacle to the effective launch of phage-based products could be production concerns (135). Regulatory approval depends on the Chemistry, Manufacturing, and Control (CMC) portion of the regulatory dossier; for phage cocktails, the CMC part must specify the manufacturing requirements for each phage as well as the procedure for mixing phages to create the finished product (135). Since regulatory bodies frequently demand that pivotal studies be conducted using products made utilizing the final process, early development of the CMC process is crucial (135).

One important factor is the cost of goods (CoGs). Phage manufacture necessitates isolated GMP manufacturing facilities because to the possibility of contamination, which is a significant capital investment that typically costs between $15 and $25 million USD (135). Furthermore, depending on the indication, those manufacturing facilities may have a very large capacity. For instance, a poultry product would need a lot of doses to treat a sizable fraction of the world’s poultry population (~14 billion birds) (135).

If profitability per animal is low, then the cost per treatment dose should be similar to that of antibiotics or vaccines. Cost reduction is possible due to economies of scale, continuous flow propagation of phages and use of agricultural by-products as fermentation media (135). However, in some cases, producers may not be willing to pay and cost-sharing schemes or regulatory incentives might be needed, e.g., to reduce broiler *Salmonella*. As reviewed by Hussain et al. (136), encapsulation and delivery technologies are critical for enhancing phage stability and enabling controlled release in the poultry gastrointestinal tract, thereby improving therapeutic efficacy in commercial poultry production. Furthermore, Shakir et al. (137) demonstrated that bacteriophage supplementation in commercial layer flocks improved growth performance and reduced disease burden by elevating immunity levels, suggesting that phage products can be economically viable when integrated into routine poultry health management.

### 6.2 Regulatory ambiguities (FDA/CVM and EMA guidelines)

The regulatory process of veterinary phage products is still incomplete and unclear (105). Veterinary phage therapies are new therapies that are covered by the EU regulatory framework for veterinary medicinal products (Regulation (EU) 2019/6), which regulates the marketing authorization process (105). The EMA has prepared the “Guideline on the quality, safety and efficacy of veterinary medicinal products specifically designed for phage therapy” which outlines the general requirements for validation of phage efficacy and safety assessment, as well as characterization of phages (105). Furthermore, a general chapter on phage therapy medicinal products for human and veterinary use has been adopted by the European Pharmacopeia (EP) under the title “Phage therapy medicinal products for human and veterinary use”, which defines quality requirements for production and control (105).

An alternative strategy has been developed in the United States where the regulatory status of phage interventions will depend on the intended use of each product—phage products can be regulated by the FDA for therapeutic use or by the USDA for food safety (105).

However, the inherent variability and potential for compositional changes in phage cocktails challenge conventional Chemistry, Manufacturing, and Controls (CMC) paradigms (105). The current regulatory environment is not conducive to the development of phage products and is considered to act as a barrier in many cases. Recent EMA and EDQM guidelines offer some flexibility to account for the biological nature of phages, but concerns remain about the feasibility of manufacturing phage-based products following existing rules for veterinary chemotherapeutants at commercially viable costs (105).

Compassionate use and magistral phage preparations under national pharmacy laws are currently the de facto pathway in Belgium and Poland (84). The lack of harmonized guidelines deters investment and prolongs time to market. A “phage registry” approach, where well-characterized phage components are pre-approved for mixing into custom cocktails, has been proposed as a pragmatic solution (84). Beyond production animals, phage therapy has also been explored in equine medicine. Sarmykova et al. (138) isolated and characterized a *Myoviridae* bacteriophage specific to *Streptococcus equi*, the causative agent of horse strangles, which is widespread in Kazakhstan and predominantly affects foals and young horses.

### 6.3 Emergence of phage resistance against cocktails

Even well-designed cocktails do not eliminate the risk of phage resistance. Long-term treatment can be undermined by bacterial counter-defenses, such as receptor mutations, abortive infection, CRISPR-Cas, and restriction-modification systems (139). Cocktails are far less prone to the emergence of resistance than monophages, however resistance is still a serious problem (9).

A promising strategy is the design of phage cocktail based on cross-resistance. Knowing the cross-resistance pattern allows phages to be clustered by resistance signatures and cocktails to be built by picking representatives of different clusters (140). This will mean that resistance to one phage will not affect another and the therapy will be more effective with less resistance building up. A cocktail of CPD4 and CPD7 phages showed high therapeutic potential against necrotic enteritis caused by *C. perfringens* in poultry (140).

Immuno-phage synergy is a paradigm change in the treatment of resistance. Pathogenic bacteria typically undergo mutations or shed essential surface structures (such as capsules, LPS, pili) to resist phage infection, at the cost of fitness (141). These compromises significantly improve host immune clearance, such as increased susceptibility to opsonophagocytosis by neutrophils, complement-dependent killing, and host chemical barriers (141). The evolutionary arms race might be changed by leveraging the dynamics of phage-induced selective pressure and immunological trade-offs, and intentionally putting pathogens into “evolutionary traps.” This strategy will convert the formidable phage resistance to a therapeutic advantage (141).

Costs of fitness of receptor mutations can help to reduce resistance, but this depends on the pathogen and the phage (139). Some of the strategies used are proactive: rotation of cocktail compositions, cocktails with organic acids or probiotics, engineering of phages to target essential bacterial receptors, and cocktails designed to anticipate bacterial resistance trajectories to guide bacterial evolution toward clinically desirable, immune-sensitive phenotypes (141). However, public and trade barriers are present for the regulatory acceptance of genetically engineered phages in food animals. In addition to these strategies, the biofilm matrix represents a further challenge to phage efficacy, as it protects embedded pathogens from lytic agents. Nevertheless, bacteriophages can penetrate the polymeric matrix and lyse biofilm-forming bacteria on food-contact surfaces, thereby improving food safety (142).

### 6.4 Standardization of titer assays

One of the seemingly trivial but important issues is the absence of a standardized plaque assay procedure in laboratories. The double-layer agar (DLA) method is still considered the standard method for enumerating bacteriophage (141). Uses two layers of agar in a petri dish: one layer for bacteria to grow, and the other layer with bacterial host and phages. Phages spread, enter bacteria and lyse them, creating visible plaques. This approach, however, must be optimized for any specific phage-host combination, and there are potential errors, including contamination, differences in bacterial growth, and operator bias (143).

For the same phage preparation, differences of up to 1 log_10_ can be obtained depending on the type of agar used, the host strain, the incubation conditions, and the type of double-layer method used. The spot assay on soft agar overlaid with MgSO_4_ gave reproducible results for phage K and 154 *S. aureus* strains, highlighting the importance of reproducible media preparation to ensure the accuracy of the results (143). The analytical specificity of the spot assay is, however, inherently limited since susceptibility of the phage remains possible without plaque formation and vice versa (143).

Phage resistance and efficacy of cocktails are influenced by phenotypic heterogeneity. To integrate phage therapy into clinical and veterinary practice, it is crucial to standardize these methods (144).

## 7 Conclusion

Bacteriophage cocktails represent a transformative approach to combating antimicrobial resistance in veterinary medicine, but their clinical success depends critically on formulation strategies that overcome physicochemical barriers encountered during storage, feed processing, and gastrointestinal transit. This review has demonstrated that encapsulation technologies—including alginate-chitosan microspheres, spray-dried powders, lipid nanoparticles, and thermosensitive hydrogels—can preserve phage infectivity when tailored to specific delivery routes and target species, yet translation from *in vitro* stability to *in vivo* efficacy remains incomplete due to overlooked factors such as gastrointestinal motility, rumen microbial activity in ruminants, and prandial state. The emergence of phage resistance, though mitigated by cocktails, requires proactive strategies including cross-resistance-guided design and exploitation of fitness trade-offs. Future progress demands species-specific physiologically based pharmacokinetic/pharmacodynamic models, regulatory harmonization, standardized titer assays, and economic incentives to accelerate commercial translation. With these advances, phage therapy can transition from a promising experimental intervention to a sustainable pillar of livestock health management, reducing antibiotic dependence and preserving antimicrobial efficacy for future generations.
